# Auditory N1 event-related potential amplitude is predictive of serum concentration of BPN14770 in fragile x syndrome

**DOI:** 10.21203/rs.3.rs-4474353/v1

**Published:** 2024-06-14

**Authors:** Jordan E. Norris, Elizabeth M. Berry-Kravis, Mark D. Harnett, Scott A. Reines, Melody A. Reese, Abigail H. Outterson, Claire Michalak, Jeremiah Furman, Mark E. Gurney, Lauren E. Ethridge

**Affiliations:** University of Oklahoma; Rush University Medical Center; Tetra Therapeutics; Tetra Therapeutics; Duke University Medical Center; Rush University Medical Center; Rush University Medical Center; Rush University Medical Center; Tetra Therapeutics; University of Oklahoma

**Keywords:** biomarker, fragile x syndrome, zatolmilast, EEG, pharmacokinetics

## Abstract

Fragile X syndrome (FXS) is a rare neurodevelopmental disorder caused by a CGG repeat expansion ≥ 200 repeats in 5′ untranslated region of the FMR1 gene, leading to intellectual disability and cognitive difficulties, including in the domain of communication. A recent phase 2a clinical trial testing BPN14770, a phosphodiesterase 4D inhibitor, showed improved cognition in 30 adult males with FXS on drug relative to placebo. The initial study found significant improvements in clinical measures assessing cognition, language, and daily functioning in addition to marginal improvements in electroencephalography (EEG) results for the amplitude of the N1 event-related potential (ERP) component. EEG results suggest BPN14770 improved neural hyperexcitability in FXS. The current study investigated the relationship between BPN14770 pharmacokinetics (PK) and the amplitude of the N1 ERP component from the initial data. Consistent with the original group-level finding in period 1 of the study, participants who received BPN14770 in the period 1 showed a significant correlation between N1 amplitude and serum concentration of BPN14770. These findings strengthen the validity of the original result, indicating that BPN14770 improves cognitive performance by modulating neural hyperexcitability. This study represents the first report of significant correlation between a reliably abnormal EEG marker and serum concentration of a novel pharmaceutical in FXS.

## Background

Fragile X syndrome (FXS) is a rare neurodevelopmental disorder caused by a repeat expansion of > 200 CGG repeats in the 5’ untranslated region of the FMR1 gene located on the X chromosome (Santoro et al., 2012; Straub et al., 2023). Clinical features of FXS include high rates of intellectual disability, anxiety, and difficulties with executive function. Cognitive features of FXS, including communication difficulties, are often cited as among the most distressing for individuals with FXS and their families with no treatment currently existing for ameliorating cognitive symptoms (Weber et al., 2019).

A recent phase 2 clinical trial assessing BPN14770 (now Zatolmilast), a first-in-class phosphodiesterase 4D (PDE4D) inhibitor, in adult males with FXS demonstrated cognitive improvements on the performance-based NIH Toolbox Cognitive Battery and in caregiver reports of language and daily functioning (Berry-Kravis et al., 2021). BPN14770 works to increase cyclic AMP (cAMP) levels by reducing phosphodiesterase activity (Gurney et al., 2019). The a priori secondary physiological measures included electroencephalography (EEG) which measured brain activity during an auditory habituation task. Specifically, the amplitude of the N1 event related potential (ERP) component was assessed and demonstrated marginal reductions suggesting improvements in neural hyperexcitability (Berry-Kravis et al., 2021). Generally, individuals with FXS exhibit increased neural responses to auditory stimuli compared to typically developed controls with the consensus that heighted neural responses to auditory stimulation reflects overall neural hyperexcitability as well as increased sensory sensitivity (Contractor et al., 2015; Ethridge et al., 2019). While BPN14770 does not target mechanisms related directly to neural hyperexcitability, a PDE4D inhibitor may support neural network organization by improving long-term potentiation through improved cyclic AMP signaling (Mierau et al., 2017).

Given the validity of the EEG findings, the N1 amplitude outcomes provide a physiological bridge between known molecular mechanisms of BPN14770 and improvements in clinical outcomes. However, reductions in usable data for ERP results reduced power to detect effects, raising the question of whether the marginal efficacy findings for the N1 ERP reflect true, but statistically underpowered, reductions in neural hyperexcitability. To confirm validity of the finding, N1 amplitude reductions were assessed against plasma BPN14770 levels from pharmacokinetic assessment.

## Methods

Participants were 30 males (age 18–41 years, M = 31.63, SD = 7.32; IQ 24.63–66.19, M = 42.78, SD = 11.6) with FXS participating in a single-center, phase 2 clinical trial assessing the efficacy and safety of BPN14770 (ClinicalTrials.gov identifier: NCT03569631, registration date: June 26, 2018). All participants or their legal guardians signed informed consent which included consent for EEG data collection. All study procedures were approved by the Institutional Review Board at Rush University Medical Center; EEG analysis and data management procedures were additionally approved by the IRB at University of Oklahoma.

### Procedure

#### Habituation Task

The auditory habituation task consisted of 150 paired 50-ms white-noise bursts with a 500 ms interstimulus-onset interval. Each stimulus train was separated by a 4,000 ms intertrial interval for a total participation time of 11.5 minutes. Event related potential (ERP) values were measured at baseline, crossover, and end of trial. The N1 amplitude was calculated for both stimuli in the habituation pair and defined as the most negative peak between 50 and 200 ms post-stimulus.

#### EEG Recording and Preprocessing

EEG data were continuously recorded and digitized at 512Hz using a 32-channel BioSemi ActiveTwo system (BioSemi) with a 5th order Bessel anti-aliasing filter at 200 Hz. Sensors were all active referenced to a driven right leg feedback loop between Cz and a central posterior ground electrode during recording. Data were inspected offline, resampled to 500 Hz, and preprocessed to remove artifacts prior to analysis using MATLAB 2018. In order, 1.) data were digitally filtered offline from 0.5 to 100 Hz with a 57–63 Hz notch, 2.) bad channels were visually inspected and interpolated with no more than ~ 5% of sensors interpolated (max of 2 channels), 3.) segments with high artifact contamination (i.e., large movement-related artifacts) were manually rejected, 4.) data were then submitted to independent component analysis (ICA) for further artifact correction, and 5.) re-referenced to the average of all channels (Delorme & Makeig, 2004).

#### Pharmacokinetics

Pharmacokinetic (PK) samples were collected at baseline and at the end of each crossover arm to confirm the study drug was present when expected (Berry-Kravis et al., 2021). The original study did not have a sufficient wash-out period and therefore the main analyses were conducted on data from period 1 between subjects. As a result, the period 1 PK value variable has 0 ng ml^−1^ BPN14770 if the participant received placebo during period 1.

#### Statistical Analysis

A Spearman rank correlation analysis was conducted to determine whether PK values correspond to a reduction in the N1 amplitude to the first stimulus in the habituation pair which demonstrated marginal significance in the original analysis of the EEG N1 ERP component. Correlations were run separately 1) including both placebo and treatment groups, and 2) including just the treatment group, to account for inherent baseline variability in N1 amplitude across participants that may skew correlation values when all individuals in the placebo group have a PK value of 0 ng ml^−1^. Additional exploratory correlations were run to assess PK relationships with the second stimulus in the habituation pair.

## Results

**Figure 1:**
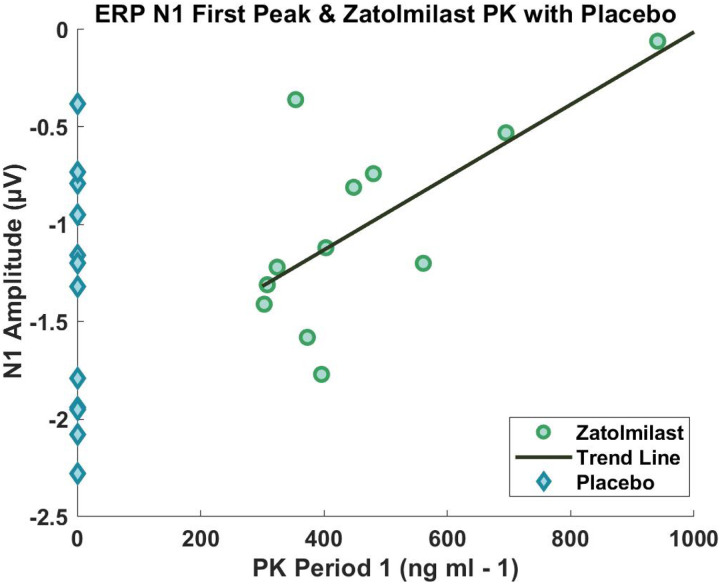
Period 1 PK – N1 Correlation for the First ERP Peak. Correlation plot for period 1 PK values and N1 amplitude. All PK values at 0 are individuals who received placebo during period 1.

Including both placebo and treatment groups in the analysis, there was a marginally significant positive correlation between the period 1 PK values and the N1 amplitude to the first stimulus (rho = .396, p = .055; N = 24 valid) but not for the second repeated stimulus (rho = .296, p = .160; N = 24 valid). The follow up correlation run on only those in period 1 who received BPN14770 confirmed the positive relationship between PK values and the N1 amplitude for stimulus 1 (rho = .608, p = .036; N = 12 valid; Fig. 1).

**Figure 2:**
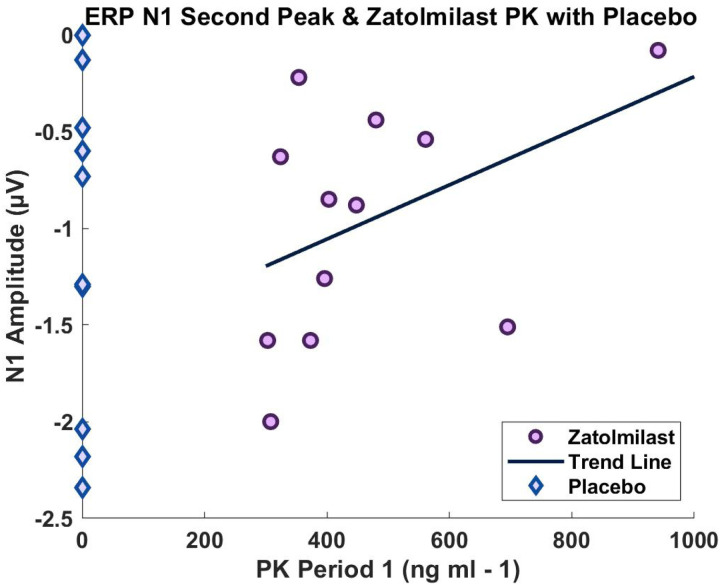
Period 1 PK – N1 Correlation for the Second ERP Peak. Correlation plot for period 1 PK values and N1 amplitude. All PK values at 0 are individuals who received placebo during period 1.

Interestingly, the correlation between the N1 amplitude of the second peak and BPN14770 PK values was marginally significant when evaluating only those who received BPN14770 during period 1 (r = .524, p = .08; N = 12 valid; Fig. 2). It is important to note that the N1 ERP amplitude is a negative value, so positive correlations indicate a reduction in N1 amplitude with increased serum concentration of BPN14770.

## Discussion

Individuals with FXS tend to exhibit increased neural responses reflected by increased ERP amplitudes to auditory stimuli compared to typically developed individuals, likely due to neural hyperexcitability (Ethridge et al., 2016; 2019; Tempio et al., 2023). The original results from the phase 2a clinical trial suggested BPN14770 reduced the ERP amplitude of the first stimulus in a habituation pair. Given the N1 is a negative-going ERP component, the positive correlation presented in the current study indicates a decrease in negative amplitude with BPN14770, which is an improvement that corresponds to a decreased neural response to the stimulus. Figure 1 demonstrates the expanded negative range of ERP values for the placebo group, highlighting the group-level shift in ERP amplitudes toward smaller values, as well as the relationship between BPN14770 serum concentration and decreases in N1 negative amplitude. The individual with the largest plasma concentration of BPN14770 shows a nearly zeroamplitude value for N1, suggesting a potential upper limit to effective dosing for this individual for maintaining the N1 amplitude within typical levels.

The current results raise confidence in the validity of the original, marginal ERP amplitude group-level result in favor of BPN14770 improvements. Confirmation of prior results lends support to the conclusion that BPN14770 reduces neural hyperexcitability during stimulus processing. Variations in the auditory N1 ERP have been associated with language and communication as well as sensory outcomes in FXS (Ethridge et al., 2016; 2019; Schmitt et al., 2020) the results have implications for language processing and may underlie the noted improvements in clinical outcomes from the original study (Berry-Kravis et al., 2021).

## Limitations

Limitations included the small sample size consistent with Phase 2 studies, expected data loss in EEG data due to movement which further reduced statistical power, and carry-over effects in placebo measures from period 2. While the correlation supports the original findings, and suggests that the effect was present but underpowered, the period 1 analyses within group reflect only 12 individuals. Nevertheless, this study represents the first report of significant correlation between a reliably abnormal EEG marker and serum concentration of a novel pharmaceutical in FXS. The N1 amplitude will be assessed as part of the ongoing phase 3 trial testing BPN14770 in a larger sample which will provide a sufficiently powered statistical model for assessing N1 amplitude improvement with BPN14770 treatment.

## Data Availability

All data generated or analyzed during this study are included in this published article. All data points reported in the analyses are presented in Figures 1 and 2.

## Abbreviations

FXS: fragile x syndrome
FMR1: fragile x messenger ribonucleoprotein 1
EEG: electroencephalography
ERP: event-related potential
PK: pharmacokinetics
NIH: National Institutes of Health
PDE4D: phosphodiesterase 4D
